# Potent molecular-targeted therapies for gastro-entero-pancreatic neuroendocrine carcinoma

**DOI:** 10.1007/s10555-023-10121-2

**Published:** 2023-07-08

**Authors:** Akira Ooki, Hiroki Osumi, Koshiro Fukuda, Kensei Yamaguchi

**Affiliations:** Department of Gastroenterological Chemotherapy, Cancer Institute Hospital of the Japanese Foundation for Cancer Research, Tokyo, Japan

**Keywords:** Gastro-entero-pancreatic neuroendocrine carcinoma, Chemotherapy, Molecular-targeted therapy, Immunotherapy

## Abstract

Neuroendocrine neoplasms (NENs), which are characterized by neuroendocrine differentiation, can arise in various organs. NENs have been divided into well-differentiated neuroendocrine tumors (NETs) and poorly differentiated neuroendocrine carcinomas (NECs) based on morphological differentiation, each of which has a distinct etiology, molecular profile, and clinicopathological features. While the majority of NECs originate in the pulmonary organs, extrapulmonary NECs occur most predominantly in the gastro-entero-pancreatic (GEP) system. Although platinum-based chemotherapy is the main therapeutic option for recurrent or metastatic GEP-NEC patients, the clinical benefits are limited and associated with a poor prognosis, indicating the clinically urgent need for effective therapeutic agents. The clinical development of molecular-targeted therapies has been hampered due to the rarity of GEP-NECs and the paucity of knowledge on their biology. In this review, we summarize the biology, current treatments, and molecular profiles of GEP-NECs based on the findings of pivotal comprehensive molecular analyses; we also highlight potent therapeutic targets for future precision medicine based on the most recent results of clinical trials.

## Introduction

Neuroendocrine neoplasms (NENs), which are characterized by neuroendocrine differentiation, can arise in various organs. NENs have generally been divided into two types based on morphological differentiation: neuroendocrine tumors (NETs) and neuroendocrine carcinomas (NECs) [1], each of which is associated with a distinct etiology, molecular profile, clinicopathological features, and treatment strategies. NECs in particular are defined by their poorly differentiated morphology and high proliferative activity [1, 2]. The transformation from a well-differentiated NET to a poorly differentiated NEC is an extremely rare event. A majority of NECs originate de novo. Alternatively, they emerge through trans-neuroendocrine differentiation of non-neuroendocrine epithelial cancers via the acquisition of genomic and epigenetic alterations during disease progression and under selective pressure, as in the case of targeted therapies within the tumor microenvironment [3–5]. Although approximately 90% of NECs originate from pulmonary organs, extrapulmonary NECs occur most commonly in the gastro-entero-pancreatic (GEP) system [6]. GEP-NEC is often diagnosed at advanced disease stages with distant metastases due to the highly aggressive behavior associated with rapid disease progression [6, 7]. Although systemic chemotherapy is the main therapeutic option for patients with metastatic GEP-NEC, the prognosis is extremely poor, with a 5-year overall survival (OS) rate of less than 5% [6]. In addition, the advancement of therapeutic strategies has seen very limited progress. Therefore, further development of novel agents is required to improve prognostic outcomes.

The existing strategy for treating GEP-NEC has been extrapolated from methods directed toward small-cell lung cancer (SCLC) that is the most predominant histology among NECs, because of the rarity of GEP-NEC and their biological similarities with SCLC [6–8]. However, pivotal studies based on comprehensive molecular analyses have shed some light on the complex molecular scenarios of GEP-NECs as well as SCLC, which have revealed some differences in their molecular profiles [6, 9]. While NECs share some genomic features, despite their different anatomical sites of origin, considerable differences also exist between sites of tumor origin [10–12]. Improved knowledge of GEP-NECs may lead to more effective therapeutic strategies, including molecular-targeted agents and immunotherapy.

In this review, we summarize the biology and current treatments of GEP-NEC, as well as the state-of-the-art knowledge of its molecular landscape that has emerged from existing comprehensive analyses. Site-specific genetic alterations are also addressed according to the organ of origin. In addition, the similarities and differences between GEP-NEC and SCLC are highlighted, where appropriate, in terms of clinicopathological and molecular features. Finally, we discuss potential therapeutic targets from both basic and clinical viewpoints.

## Clinicopathological and molecular features of GEP-NEC

NETs and NECs are distinct entities with widely differing etiologies, clinicopathologies, and genomic profiles.

### Classification of neuroendocrine neoplasms

**Fig. 1:**
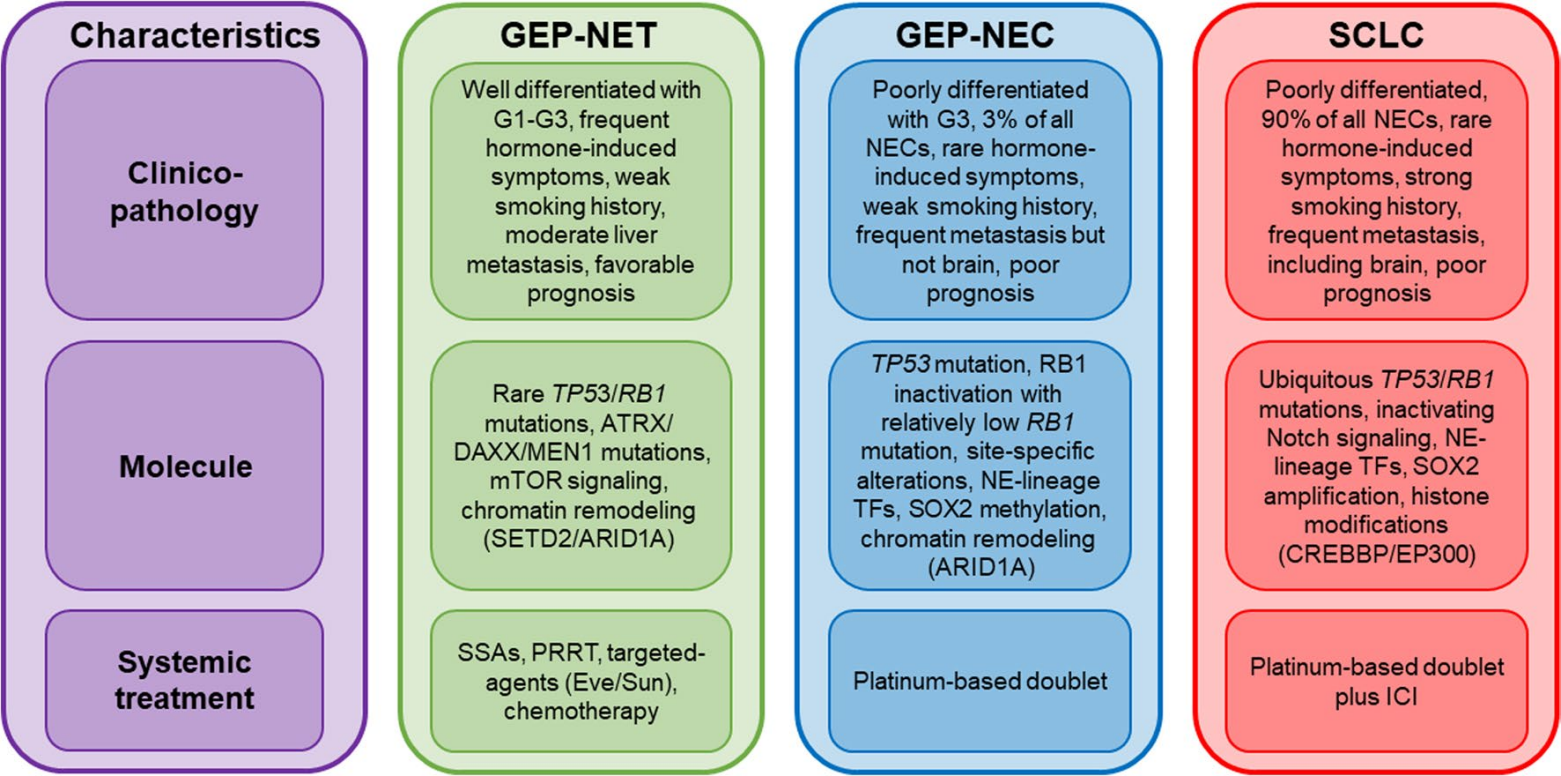
Characteristics and treatment of GEP-NET, GEP-NEC, and SCLC. For patients with GEP-NET, synthetic somatostatin analogs (SSAs) are used due to their favorable biology, including relatively low Ki-67, slow growth, and positive somatostatin receptor (SSTR) expression. Chemotherapy regimens recommended for advanced GEP-NETs and G3 GEP-NETs include streptozotocin-based, temozolomide-based, and platinum-based treatments. Molecular-targeted agents such as everolimus (Eve) and sunitinib (Sun) are also available, with Sun currently approved for pancreatic NETs only. Peptide receptor radionuclide therapy (PRRT) is an option for patients with progressive NETs expressing SSTR after first-line therapy. For patients with GEP-NEC, the standard first-line regimen remains cisplatin plus etoposide or cisplatin plus irinotecan. For patients with SCLC, the standard regimen consists of platinum-based chemotherapy combined with an immune checkpoint inhibitor (ICI) such as atezolizumab or durvalumab. NE, neuroendocrine; TFs, transcription factors

While GEP-NENs share a neuroendocrine phenotype, they are heterogenous malignancies that can originate from different anatomical sites. Based on grading of the Ki-67 proliferation index of the World Health Organization (WHO) classification in 2010, GEP-NENs can be categorized as low grade (G1), intermediate grade (G2), or high grade (G3), with Ki-67 values of < 3%, 3–20%, and > 20%, respectively [13]. Furthermore, the WHO 2017 and 2019 classifications separated the G3 GEP-NENs into well-differentiated G3 NETs and poorly differentiated G3 NECs based on their morphological differentiation as two distinct entities in terms of prognostic and molecular features [2, 10, 12–14]. Consequently, GEP-NENs are now classified as well-differentiated G1–G3 NETs and poorly differentiated G3 NEC based on proliferative grading and differentiation (Table 1 and Fig. 1). GEP-NECs are histopathologically subdivided into small-cell NEC (SCNEC) and large-cell NEC (LCNEC) [15].

**Table 1:** WHO 2019 classification for GEP-NENs

Terminology	Differentiation	Grade	Ki-67 index	Mitotic rate
NET, G1	Well differentiated	Low	< 3%	< 2
NET, G2	Well differentiated	Intermediate	3–20%	2–20
NET, G3	Well differentiated	High	> 20%	> 20
NEC (SCNEC)	Poorly differentiated	High	> 20%	> 20
NEC (LCNEC)	Poorly differentiated	High	> 20%	> 20
MiNEN	Well or poorly differentiated	Variable	Variable	Variable

Abbreviations: WHO, World Health Organization; GEP, gastro-entero-pancreatic; NEN, neuroendocrine neoplasm; NET, neuroendocrine tumor; NEC, neuroendocrine carcinoma; SCNEC, small cell neuroendocrine carcinoma; LCNEC, large cell neuroendocrine carcinoma; MiNEN, mixed neuroendocrine/nonendocrine neoplasm, defined as the co-existence of NENs and non-neuroendocrine tumors, with at least 30% of each component; mitotic rates, the number of mitoses/2 mm2; Ki-67 proliferation index value, percentage of at least 500 tumor cells in the regions of the highest nuclear labeling using MIB1 antibody

### Clinicopathological features of GEP-NEC

The clinicopathological features of NECs and NETs are shown in Table 2 and Fig. 1. Morphologically, NECs present with solid “sheet-like” growth, partial or complete loss of cyto-architecture, irregular nuclei, less secretory granules, high mitoses, and abundant necrosis [2]. The expression of neuroendocrine markers is extremely limited in NECs [14, 16–18]. Although G3 NENs are defined by a Ki-67 proliferation index > 20%, NECs usually have a high Ki-67 index value of ≥ 55% compared to G3 NETs with a Ki-67 index of 21–50% [7, 14, 19, 20].

**Table 2:** Clinicopathological features of GEP-NENs [1, 2, 6–8, 12–14, 16–39]

Characteristics	NEC	NET
Proportion of GEP-NENs	10%	90%
Proportion of all extrapulmonary NECs	One-third	-
Incidence trend	Increasing	Increasing
Association with smoking history	Weak	Weak
Sex	Male > female	Male = female
Median age	60–70 years	60 years
Location of primary tumor	Colorectum, pancreas, and esophagus-stomach	Rectum, pancreas, and stomach in Asia, and small intestine and appendix in Europe
Diagnosis at a metastatic stage	60 to 85%	20%
Most common sites of metastases	Lymph node, liver, and peritoneum	Liver
Brain metastases	Rare	Rare
Prognostic outcomes	Poor (5–8 months)	Good (20–53 months), 18–40 months for G3 NET
Survival trend	Improving	Stable
Neuroendocrine differentiation markers	Diffuse positive (INSM1 and Syn) and focal/faint positive (CgA)	Strong positive (INSM1, Syn, and CgA)
SSTRs expression	Weak to absent	Strong
Hormone	Weak to absent	Strong
Ki-67 index	High (typically ≥ 55%)	Low (typically < 55%)
Mitosis	High	Low
Morphology	Sheet-like architecture, abundant necrosis, and high nuclear/cytoplasm ratio	Organoid or nested architecture, including trabecular, glandular, or solid patterns; minimal necrosis; round nuclei; and finely granular cytoplasm
Morphology of SCNEC	Similar to SCLC, which is composed of relatively small cells with a high nuclear/cytoplasmic ratio, tightly packed fusiform nuclei, hyperchromatic and finely granular chromatin, and inconspicuous nucleoli	-
Morphology of LCNEC	Round to polygonal large cells with moderate amounts of cytoplasm, more rounded nuclei, vesicular chromatin, and prominent nucleoli	-
Proportion of small cell versus large cell morphology	40% vs. 60%	-
Predominant small cell morphology	Esophagus, anal, and gallbladder	-
Predominant large cell morphology	Small intestine, colorectum, stomach, liver, biliary tract, and pancreas	-

Abbreviations: GEP, gastro-entero-pancreatic; NEN, neuroendocrine neoplasm; NET, neuroendocrine tumor; NEC, neuroendocrine carcinoma; SCNEC, small cell neuroendocrine carcinoma; LCNEC, large cell neuroendocrine carcinoma; CgA, chromogranin A; Syn: synaptophysin; INSM1, insulinoma-associated protein 1; SSTR, somatostatin receptor

Approximately 10% of all NECs originate from the extrapulmonary organs, among which one-third develop in the GEP system [6, 8, 17]. The main primary locations of GEP-NECs have been identified as the colorectum, pancreas, and esophagus-stomach [6, 7, 14, 21], and large-cell morphology is the predominant subtype, except for a few tumor sites, such as the esophagus, the gall bladder, and the anal canal, in contrast to pulmonary NECs, which exhibit the highest percentage of small-cell morphologies (95%) [6]. Hormone-induced symptoms are rare [7, 16]. NECs are aggressive phenotypes that are identified as metastatic diseases in 60–85% of cases at the time of diagnosis [6–8, 14, 17, 21, 22]. Metastases are often found in the liver, lymph node, or peritoneum, and the incidence of brain metastases is considerably lower in GEP-NEC compared with pulmonary NEC [7, 8, 14, 22–24]. Although improved survival for GEP-NECs has been reported [17, 25, 26], the prognosis of patients with metastatic NECs is still unfavorable, with a median OS of 5–8 months [6, 8, 17]. The survival rate for GEP-NEC patients has been found to be worse than that for G3 NETs, even among G3 NENs [1, 12, 19, 27, 28, 40, 41]. Therefore, further development of novel therapies for GEP-NECs is warranted.

### Aberrant molecular profiles of GEP-NEC

Developments in high-throughput genomic technologies have led to a better understanding of the molecular profiles of GEP-NEC compared with SCLC or GEP-NET (Table 3 and Fig. 1). SCLC is the most predominant histology among pulmonary NECs [6], and it is characterized by ubiquitous inactivating mutations in both *TP53* and RB transcriptional corepressor 1 (*RB1*) [42–46]. NEC can develop as a result of lineage plasticity in response to selective pressure from targeted therapies, a process that is also associated with the loss of TP53 and RB1 [3, 47–50]. Genetically engineered mouse models demonstrated that inactivation of Trp53 and Rb1 induced lineage plasticity by converting from an epithelial phenotype to a NEC phenotype [5, 48, 51]. Genomic aberrations in *TP53* and *RB1* have also been observed in GEP-NEC at frequencies ranging from 57 to 89% and 9 to 46%, respectively [10–12, 29, 52–57], thus supporting the idea that the NEC phenotype shares part of the genetic processes of tumor evolution, regardless of the anatomic site of tumor origin [5, 30]. Chromothripsis is a single catastrophic event in the genome that is associated with *TP53* mutation in GEP-NEC [10]. Although *RB1* mutations emerge at a much lower rate in GEP-NEC compared to SCLC, the RB1 pathway appears to be suppressed by other mechanisms, such as copy number alterations affecting RB1, silencing of p16 via promoter methylation of cyclin-dependent kinase inhibitor 2A (*CDKN2A*), and amplifications of *MYC* or cyclin E1 (*CCNE1*) as an RB1 antagonist [10, 12, 45, 56, 58, 59]. In addition, a loss of RB1 protein expression has been reported in 33–80% of GEP-NEC cases [20, 60–63]. Thus, the loss of TP53 and RB1 pathways is a prerequisite for both the pathogenesis and lineage plasticity of NEC, but these alterations are likely to be insufficient to drive lineage reprogramming of the NEC phenotype [3, 5, 56, 64, 65]. Additional oncogenic factors are needed to transform normal epithelial cells into SCNEC [5, 56].

**Table 3:** Dysregulated genetic alterations in GEP-NEC, GEP-NET, and SCLC

		GEP-NEC [10–12, 29, 54, 55, 57]	GEP-NET [12, 29, 52, 67, 68]	SCLC [42–46]
Molecules	Signaling pathway	Frequency of genetic aberrations (%)		
Cell cycle				
TP53	Regulator of cell cycle	57–89	3–15	86–98
RB1	Regulator of cell cycle	9–46	0–33	67–91
ATM	Regulator of cell cycle	0–35	2–3	1–3
CDKN2A	Regulator of cell cycle	3–19	0–4	2–5
CCNE1	Regulator of cell cycle	0–12	0–4	1–8
RTK/RAS/MAPK and PI3K				
BRAF	MAPK/PI3K pathway	7–20	0–4	0–1
KRAS	MAPK/PI3K pathway	8–30	0–3	0–4
PIK3CA	PI3K pathway	3–9	0–4	3–5
mTOR	PI3K pathway	1	3–7	2
TSC2	PI3K pathway	4	4–9	2
PTEN	PI3K pathway	2	7	6–9
EGFR	RTK	0–3	0–1	0–3
ERBB2	RTK	3–5	0–1	0–1
ERBB3	RTK	2–6	0–4	3–6
FGFR1	RTK	0	0–4	1–6
VHL	Angiogenesis	0	1–7	0–1
Cell adhesion and proliferation				
CTNNB1	Wnt/β-catenin pathway	6–9	0–4	0
APC	Wnt/β-catenin pathway	9–29	0–11	0–3
MYC	Transcription factor	8–51	1–38	0–16
SMAD4	TGFβ pathway	5–9	0–8	0–2
SMAD2	TGFβ pathway	1	1–8	0
PTCH1	Hedgehog pathway	2–3	0–8	0–5
Cell differentiation				
SOX2	Transcription factor	0	0	0–27
NOTCH1	Transcription factor	5–10	1–8	11–13
DLL3	Notch pathway	0	0	0–3
FBXW7	Ubiquitin ligase	4–12	0	1–4
YAP1	Co-transcription factor	1	0	0
Chromatin modification				
DAXX	Altered telomere length	0–1	14–25	0–2
ATRX	Altered telomere length	5	7–19	1–8
MEN1	Histone modifiers	1	10–44	0–1
KMT2D	Histone modifiers	10–12	1–8	18
KMT2C	Histone modifiers	4	5–8	10
CREBBP	Histone modifiers	3–6	0–4	3–15
EP300	Histone modifiers	3	0–1	5–13
EZH2	Histone modifiers	1	0–8	1
ARID1A	SWI/SNF	35–40	2–58	3
SMARCA4	SWI/SNF	5	0	1–4
Immune checkpoint inhibitors				
MSI-H [12, 27, 52, 54, 66, 69–75]	DNA mismatch repair	0–13	0	0–2

Also referred to cBioPortal for Cancer Genomics (https://www.cbioportal.org/)

Abbreviations: GEP, gastro-entero-pancreatic; NET, neuroendocrine tumor; NEC, neuroendocrine carcinoma; SCLC, small cell lung cancer; RTK, receptor tyrosine kinase; SWI/SNF, switch/sucrose nonfermentable

In addition to the mutations of *TP53* and *RB1*, other frequently mutated genes in GEP-NECs are *KRAS*, *BRAF*, adenomatosis polyposis coli (*APC*), *CCNE1*, *CDKN2A*, Notch receptor 1 *(NOTCH1*), F-box and WD repeat domain containing-7 (*FBXW7*), catenin beta 1 (*CTNNB1*), and phosphatidylinositol-4,5-bisphosphate 3-kinase catalytic subunit alpha (*PIK3CA*)/phosphatase and tensine homolog (*PTEN*) [10–12, 29, 54, 55, 57] (Table 3). The ataxia telangiectasia-mutated (*ATM*) gene was subject to frequent copy number losses, whereas the *MYC* gene was frequently amplified [12]. In a systematic review of 41 studies assessing the molecular features of GEP-NECs, common alterations were observed in the signaling cascades of the mitogen-activated protein kinase (MAPK), p16/cyclin D1/RB1, and Notch pathways [66]. Notably, these molecular features had limited similarities to SCLC. In a clustering analysis of the COSMIC single-base substitution signatures, GEP-NECs exhibited no smoking-related signatures that are representative in SCLC, indicating distinct mutational processes between GEP-NEC and SCLC [10].

SCLC has typically been classified into four molecular subtypes based on the expression status of distinct neuroendocrine-lineage-specific transcription factors, including achaete-scute family bHLH transcription factor 1 (ASCL1), neuronal differentiation 1 (NEUROD1), POU class 2 homeobox 3 (POU2F3), and yes1-associated transcriptional regulator (YAP1) [76]. These subtypes have distinct features of a neuroendocrine phenotype, epithelial-mesenchymal transition, a tumor immune microenvironment, expression profiles, and therapeutic vulnerabilities [76, 77]. In a DNA-binding motif enrichment analysis of the reprogrammed SCNEC, motifs corresponding to ASCL1, NEUROD1, and NKX homeodomain, including NKX2.5 were hyper-accessible transcription factor binding regions [5]. Similarly, in GEP-NECs, ASCL1, NEUROD1, POU3F2, YAP1, and NKX2-5 have been identified as potential master regulators of neuroendocrine lineage reprogramming [56, 58, 78]. However, the expression pattern has been found to differ from that of SCLC [56]. Recently, special attention has been paid to SRY-box transcription factor 2 (SOX2), which acts not only as a transcriptional target of ASCL1 [79], but also as a prominent transcription factor that promotes pluripotency in embryonic stem cells [80] and cancer stem cells [81–83]. The *SOX2* gene is recurrently amplified in SCLC [43] and has been implicated in the phenotypic switch as lineage plasticity [48]. In GEP-NECs, SOX2 is frequently overexpressed via the hypermethylation of its promoter region [10]. Thus, specific transcription factors can govern neuroendocrine differentiation and transformation in GEP-NECs.

Epigenetic aberrations are among the most oncogenic processes in SCLC [42] and GEP-NECs [11]. Histone-modifying genes, including lysine methyltransferase 2D (*KMT2D*), lysine methyltransferase 2C (*KMT2C*), CREB-binding protein (*CREBBP*), and E1A-binding protein p300 (*EP300*), have been shown to be frequently altered in SCLC [42], and mutations of these genes are largely mutually exclusive in GEP-NEC [11, 56, 58] (Table 3 and Fig. 1). In addition, alterations of switch/sucrose nonfermentable (SWI/SNF) chromatin remodeling genes, including AT-rich interaction domain 1A (*ARID1A*), are more common in GEP-NEC and GEP-NET, but rare in SCLC [10–12, 42, 52]. Therefore, epigenetic regulation may be a viable therapeutic target in GEP-NECs.

Although GEP-NECs share some genomic alterations characterized by neuroendocrine lineage regardless of different primary organ sites, they also have organ-specific mutational signatures [10]. A pivotal comprehensive molecular analysis has shown the different genomic features and methylation statuses between pancreatic NECs and non-pancreatic NECs in GEP systems [10]. Compared to pancreatic NECs, non-pancreatic NECs have a larger number of structural variations and nonsynonymous mutations [10]. Regarding the Notch signaling pathway, which acts as a tumor suppressor and master regulator of neuroendocrine differentiation in SCLC [42], aberrations of Notch family genes were frequently observed in non-pancreatic NECs, especially esophageal NECs [10, 11, 57]. Importantly, GEP-NECs exhibit key genetic aberrations identical to the non-neuroendocrine carcinomas arising in the same sites, such as *BRAF* and *APC* mutations in colorectal NECs [11, 12, 29, 45, 57, 84–88], *KRAS* mutation in colorectal and pancreatic NECs [9, 11, 12, 20, 29, 45, 55, 57], *NOTCH1* mutation in esophageal NECs [11, 12, 57], and E74-like ETS transcription factor 3 (*ELF3*) mutation in ampullary NECs [10, 89] (Table 4 and Fig. 1), suggesting the hypothesis that GEP-NECs and non-neuroendocrine carcinomas originate from common clonal precursors in the same organ [3, 10, 90].

**Table 4:** Genetic alterations according to the organ sites of GEP-NECs

Primary organ sites	Incidence among GEP-NECs (%) [6]	Frequency of genetic aberrations (%)									Special comments
		TP53	RB1	KRAS	BRAF	ERBB2	APC	MYC	NOTCH1	BRCA2	
Pancreas [9, 11, 12, 20, 45]	20	69–100	61–89	7–49	20–23	8	3–15	46	8	0	Almost half of genetic alterations in pancreatic NECs are related to pancreatic ductal adenocarcinoma, which include *TP53*, *KRAS*, *BRAF*, *APC*, *CDKN2A*, *ARID1A*, and *PIK3CA*/*PTEN* [9, 11, 12, 20, 45, 55, 91]. Pancreatic NECs are molecularly classified into two subtypes: (1) “ductal-type” with mutations of *KRAS* and *TP53*, loss of *RB1*, CpG island methylator phenotype, and overexpression of transcription factors, such as SOX2, ASCL1, NKX2-1, EZH2, and E2F1 and (2) “acinar-type” with aberrant Wnt/β-catenin signaling, mutation of *CDKN2A*, and overexpression of transcription factors, such as PTF1A, GATA4, and NR5A2, and RBPJ [10]
Stomach [12, 57]	12	69–90	18–44	1–19	6–11	3–6	3–12	0–44	5–6	5–6	The frequently altered genes in gastric NECs were *TP53*, *ARID1A*, *RB1*, and *KDM5A* [12, 92]. Gastric NECs showed a higher frequency of *APC* mutations than pulmonary NECs and a lower frequency of *KRAS* and *BRAF* mutations than colorectal NECs [92]. Although the *ERBB2* gene is amplified in approximately 20% of all gastric adenocarcinomas [93], the rate is only 5% in gastric NECs [12, 94]. ERBB2 expression is consistently absent in gastric NECs, regardless of the *ERBB2* amplification status [94]. Genetic aberrations of the Wnt/β-catenin pathway were prevalently identified in gastric NECs [57]
Esophagus [11, 12, 57, 58]	11	85–93	30–41	0–6	0	0	3–11	0–83	11–31	3–11	Significantly mutated genes are *TP53*, *RB1*, and *NOTCH1*, acting as putative tumor suppressors [11, 12, 57, 58]. RB1 is universally disrupted by other multiple mechanisms in addition to its mutation [58]. Mutations of the *NOTCH1* gene were more frequently observed in esophageal NECs compared to the other GEP-NECs [11, 12, 57], and the Notch signaling pathway is constitutively suppressed by the downregulation of Notch receptors and effectors, as well as the overexpression of Notch antagonists, such as DLL3 [57, 58]. In addition, esophageal NECs exhibit a lower frequency of somatic copy number variants that are frequently altered in conventional esophageal cancers, such as *CDKN2A* and *CCND1* in the cell cycle pathway and *ERBB2* in the RTK pathway [95]. Multi-omics analysis of esophageal NECs revealed two molecular subtypes based on expression patterns regulated by ASCL1 and NEUROD1 neuroendocrine-lineage transcription factors [58]. These two subtypes were highly similar to the corresponding SCLC subtypes, and *MYC* amplification was significantly enriched in the NEUROD1-proficient subtype. Collectively, esophageal NECs have similar genomic alterations, transcriptome features, and molecular subtypes to SCLC, but they are quite different from conventional esophageal cancers
Colorectum [11, 12, 29, 45, 53, 57, 84–86]	38	43–80	18–34	17–53	15–59	5–7	37–70	5–62	0	0–2	Colorectal NECs show features of NECs, such as mutation of the *TP53* gene, copy number losses of *RB1*, *ARID1A*, and *ATM* genes, amplification of the *MYC* gene, and overexpression of p16 and the BCL2 [12, 54, 59, 63]. Colorectal NECs also have a similar mutational repertoire to CRC [88], with high mutation rates of CRC-associated genes, including the *KRAS*, *BRAF*, *APC*, *FBXW7*, and *SOX9* genes [12, 29, 57]. *APC* mutation is exhibited at a much higher proportion in colorectal NECs than in the other GEP-NECs [45, 57]. The majority of colorectal NECs have been found to harbor genetic alterations in RAS/MAPK and PI3K pathways, akin to CRC [57]. There is also a distinct methylome between colorectal NEC and CRC as epigenetic events, suggesting a different gene expression profile and biological behavior [88]
Small intestine [57]	5	86	43	14	14	14	0	0	14		Small intestinal NECs are almost exclusive to the ampullary region [96]. *ELF3* is a significantly mutated driver gene in ampullary carcinomas and NECs [10, 89]. *CTNNB1* encodes a β-catenin protein that acts as an essential part of the Wnt/β-catenin signaling pathway, and *CTNNB1* mutations were observed at a high frequency in small intestinal NECs [57]
Biliary tract [12, 97]	6	73	27	7	0	0	0	7	13	13	In a comprehensive genomic analysis between NECs and conventional cancers of gallbladder, the average number of mutations were lower in gallbladder NECs [97]. *RB1* and *NAB2* genes were more significantly mutated in gallbladder NECs, whereas mutations of the *APC*, *BRAF*, and *ERBB2* genes were observed exclusively in conventional cancers. Genes carrying somatic single-nucleotide variants were enriched mainly in the Notch, Wnt/β-catenin, Hippo, and RTK/RAS oncogenic signaling pathways. Amplifications of *MYC* or *CCNE1* genes acting as RB1 antagonists were also detected in NECs. A study including 34 gallbladder NECs showed loss of RB1 and concomitant overexpression of p16 in 74% of all cases [98], indicating a driver role for the RB1 pathway in gallbladder NECs

In pulmonary NEC, LCNECs are genetically more heterogeneous than SCNECs, with frequent inactivation of both TP53 and RB1 [42]. In GEP-NECs, mutations of the *RB1* gene were more prevalent in SCNECs than in LCNECs, whereas structural variants in the *RB1* gene were more frequent in LCNECs, indicating different mechanisms of RB1 inactivation [10]. However, the genetic profiles were highly concordant between GEP-SCNECs and GEP-LCNECs [9, 12, 57].

Both the alpha-thalassemia/mental retardation syndrome X-linked (ATRX) and death-domain-associated protein (DAXX) play a role in chromatin remodeling at telomeres and other genomic sites [99], and the multiple endocrine neoplasia type 1 (MEN1) interacts with DNA damage repair, chromatin remodeling, telomere alteration, and the phosphatidylinositol-4,5-bisphosphate 3-kinase (PI3K)/mechanistic target of rapamycin kinase (mTOR) pathway [67]. In pancreatic NENs, most NETs harbor genetic mutations of *MEN1*, *ATRX*, or *DAXX*, but mutations of the *TP53* and *RB1* genes are not observed frequently in NECs [2, 7, 9–11, 13, 67, 68, 100]. Among G3 GEP-NENs, G3 NETs share common genetic and epigenetic alterations with a hallmark of G1/G2 NETs, but not of NECs [10, 12, 52, 101]. In fact, G3 NET has frequent mutations in *MEN1*, *ATRX*, or *DAXX*, but extremely rare mutations in *TP53*, *RB1*, and *KRAS* [9, 12, 13, 20, 52, 61, 62, 102]. The frequency of mutations is substantially higher in GEP-NECs than in GEP-NETs [10, 13, 55].

Collectively, GEP-NECs adopt a subset of genomic and epigenomic characteristics of SCLCs, but some key molecular alterations are organ specific, even in the GEP system. In addition, distinct molecular profiles between NECs and NETs support the notion that NECs are not derived directly from NETs [3, 9].

## Current treatment of patients with GEP-NEC

GEP-NEC patients are often diagnosed at advanced stages and are not eligible for curative treatment. For such patients, systemic treatment is the main therapeutic option for prolonging survival and improving their symptoms and quality of life. The current therapeutic strategies for GEP-NENs differ according to NET and NEC subtypes (Fig. 1). Systemic treatment for NETs includes four types of treatment: (1) synthetic somatostatin analogs (SSAs), (2) peptide receptor radionuclide therapy (PRRT), (3) molecular-targeted agents, and (4) cytotoxic agents. For NECs, cytotoxic chemotherapy is the only established treatment [31, 103].

Therapeutic strategies for patients with GEP-NECs originated from those designed for SCLCs because of their close tumoral entity and the rarity of GEP-NEC [8, 31, 103]. Platinum-based chemotherapy is recommended as a first-line treatment extrapolation from SCLC [8, 24, 31, 103]. The impact of cytotoxic chemotherapy on GEP-NECs has mostly been evaluated by retrospective studies (Table 5). The treatment efficacy of platinum-based chemotherapy is generally modest, with a reported overall response rate (ORR) of 14–75% and a median progression-free survival (PFS) of 1.8–8.9 months. The median OS is approximately 12 months [14, 22, 104–106]. In a national cancer database study comprising 1861 patients with GEP-NECs, patients treated with palliative chemotherapy had significantly improved survival outcomes, compared to those who did not receive this treatment, with median OS of 11.2 months and 1.7 months, respectively (hazard ratio [HR], 0.43; 95% confidence interval [CI], 0.39–0.48) [22]. Similar results were observed in the NORDIC NEC study [14]. Of note, the Ki-67 proliferation index was a predictive marker for platinum-based chemotherapy (median ORR, 15% for patients with Ki-67 < 55% and 42% for those with Ki-67 ≥ 55%). This finding also supports the idea that platinum-based chemotherapy has limited efficacy for G3 NETs, whose Ki-67 index values are usually less than 55% [7, 19, 20]. Although the prognostic difference between organ sites remains controversial [14, 104], there was no difference found between cisplatin (CDDP) and carboplatin among platinum compounds in terms of treatment efficacy [14]. The two most commonly used chemotherapy regimens are etoposide (ETP) plus CDDP (EP) and irinotecan (CPT-11) plus CDDP (IP) [8, 31, 103]. In a phase III TOPIC-NEC trial of EP versus IP for GEP-NEC in a first-line setting, superiority was not demonstrated because of the median OS with an HR of 1.04 (95% CI: 0.79–1.37) [104]. Thus, both EP and IP remain standard first-line regimens.

**Table 5:** Clinical trials of cytotoxic chemotherapy for GEP-NEC

Study	Primary sites	No. of pts	Regimen	ORR (%)	mPFS (months)	mOS (months)	Ref
First-line cytotoxic chemotherapy							
Retro	Any	41	CDDP + ETP	42	8.9	15	[107]
Pros	Any	18	CDDP + ETP	67	8	19	[108]
Retro	HBP	21	CDDP + ETP	14	1.8	5.8	[109]
Retro	GEP	19	CBDCA + ETP	47	7.0	12.7	[110]
Retro	Extrapulmonary	106	CBDCA + ETP	48	6.0	11.5	[111]
Retro	GEP or UK	21	Platinum + ETP	52	7	16	[112]
Retro	GEP	113	Platinum + ETP	35	5.0	16.4	[19]
Pros	GEP	152	Platinum + ETP	50	6.2	11.6	[21]
Retro	GEP	236	Platinum + ETP	27	4.6	13	[83]
Phase II	Extrapulmonary	78	CDDP + ETP + PTX	53	7.5	14.5	[113]
Phase II	Any	20	CDDP + CPT-11	58	4	-	[114]
Retro	Gastric	22	CDDP + CPT-11	75	7.1	22.6	[115]
Retro	Extrapulmonary	28	CDDP + CPT-11	64	6.4	16	[116]
Retro	Esophageal	12	CDDP + CPT-11	50	4.0	12.6	[117]
Retro	GEP	16	CDDP + CPT-11	57	5.5	10.6	[118]
Retro	Extrapulmonary	28	CDDP + CPT-11	46	3.7	11.7	[119]
Phase II	GEP	40	CDDP + CPT-11 + Oct-LAR	45	5.7	12.9	[120]
Retro	Pancreatic	29	Platinum-based regimen	37	-	10.1	[121]
Retro	GEP	160	CDDP + CPT-11	50	5.2	13.0	[82]
		46	CDDP + ETP	28	4.0	7.3	
Retro	GEP	252	Platinum-based regimen	31	4	11	[14]
		129	CDDP + ETP	31	4	12	
		67	CBDCA + ETP	30	4	11	
		28	CBDCA + VCR	44	4	10	
rPhase II	GEP	33	CDDP + ETP	42	6.4	11.3	[122]
		33	CDDP + CPT-11	42	5.8	10.2	
Phase III	GEP	84	CDDP + ETP	55	5.6	12.5	[81]
		86	CDDP + CPT-11	53	5.1	10.9	
Retro	GEP	11	FOLFIRI	64	6.5	13.0	[123]
Second- or later-line cytotoxic chemotherapy							
Pros	GEP	72	FOLFIRI	24	2.9	5.9	[21]
Retro	GEP	19	FOLFIRI	31	4	18 from diagnosis	[124]
Retro	GEP	5	FOLFIRI	40	5.8	11	[125]
Pros	GEP	33	FOLFOX	16	2.3	3.9	[21]
Retro	Any	20	FOLFOX	29	4.5	9.9	[126]
Phase II	Any	13	XELOX	23	4	5	[127]
Retro	GEP or UK	28	TEM	0	2.4	3.5	[128]
Retro	Any	25	TEM ± cape ± Bev	33	6	22	[129]
Retro	GEP	12	TEM + cape	8	3.3	4.6	[130]
Retro	GEP	46	TEM + cape or TEM mono	26	2	13.1	[87]
Retro	GEP	84	TEM-based or taxan-based	18	-	19 from 1^st^ line	[14]
Retro	GEP	10	AMR	20	2.6	5.0	[131]
Retro	GEP	13	AMR	39	3.6	7.2	[132]
Retro	GEP	19	AMR	19	3.8	7.7	[133]
Retro	GEP	16	AMR	6	2.9	13.8	[134]
Retro	Any	30	TPT	7	2.1	4.1	[135]
Retro	GEP	22	TPT	0	2.1	3.2	[136]
Phase II	GEP	23	Lipotecan	0	1.8	4.3	[137]
rPhase II	Extrapulmonary	29	nal-IRI/5-FU	10	3	9	[138]
		29	DTX	10	2	5	

Some of studies included heterogeneous populations of well-differentiated G3 NET and poorly-differentiated G3 NEC

Abbreviations: GEP, gastro-entero-pancreatic; NEC, neuroendocrine carcinoma; HBP, hepatobiliary pancreatic; UK, unknown; ORR, overall response rate; mPFS, median progression-free survival; mOS, median overall survival; Pts, patients; Ref, reference; Retro, retrospective study; Pros, prospective study; rPhase II, randomized phase II; Oct-LAR, octreotide long acting release; CDDP, cisplatin; CBDCA, carboplatin; CPT-11, irinotecan; ETP, etoposide; VCR, vincristine; PTX, paclitaxel; FOLFOX, 5-fluorouracil + leucovorin + oxaliplatin; FOLFIRI, 5-fluorouracil + leucovorin + irinotecan; XELOX, capecitabine + oxaliplatin; TEM, temozolomide; Bev, bevacizumab; Cape, capecitabine; Mono, monotherapy; AMR, amrubicin; TPT, topotecan; DTX, docetaxel; nal-IRI, nanoliposomal irinotecan; Lipotecan, a novel camptothecin analog

Unfortunately, there is no standard chemotherapy for a second-line setting. In a systematic review and meta-analysis of second-line treatment in 582 patients with advanced extrapulmonary NEC, the ORR and median PFS were 18% and 2.5 months, respectively [139]. Similarly, the NORDIC NEC study showed an ORR of 18% in second-line chemotherapy for 84 patients with GEP-NEC [14]. Several chemotherapeutic agents have been proposed based on the results of small studies conducted on the second or later line (Table 5). Special attention should be paid to these results because of heterogeneous populations, including cases of well-differentiated G3 NET and poorly differentiated G3 NEC, which show that 5-fluorouracil (5-FU), oxaliplatin, and CPT-11 are likely to have antitumor activity in cases of GEP-NEC. Therefore, FOLFIRINOX, consisting of these three agents, may be a promising regimen, as demonstrated in cases of pancreatic cancer [140]. A randomized phase II trial to compare the efficacy and safety of first-line FOLFIRINOX treatment versus EP in GEP or unknown primary NECs is ongoing (NCT04325425). It should also be noted that temozolomide is active in pancreatic NETs [8, 31, 103], and a combination of capecitabine and temozolomide (CAPTEM) demonstrated a high ORR and long PFS compared to temozolomide alone [141]. In a multicenter retrospective review of 130 patients with G3 GEP-NENs, including NEC (35% of the study population), the ORR of the CAPTEM regimen was 26% [142]. Currently, randomized phase II trials of CAPTEM versus platinum plus ETP in the first-line setting (NCT02595424) and CAPTEM or FOLFIRI as a second-line therapy (NCT03387592) in GEP-NECs are ongoing.

## Potent molecular-targeted therapy for patients with GEP-NEC

**Fig. 2:**
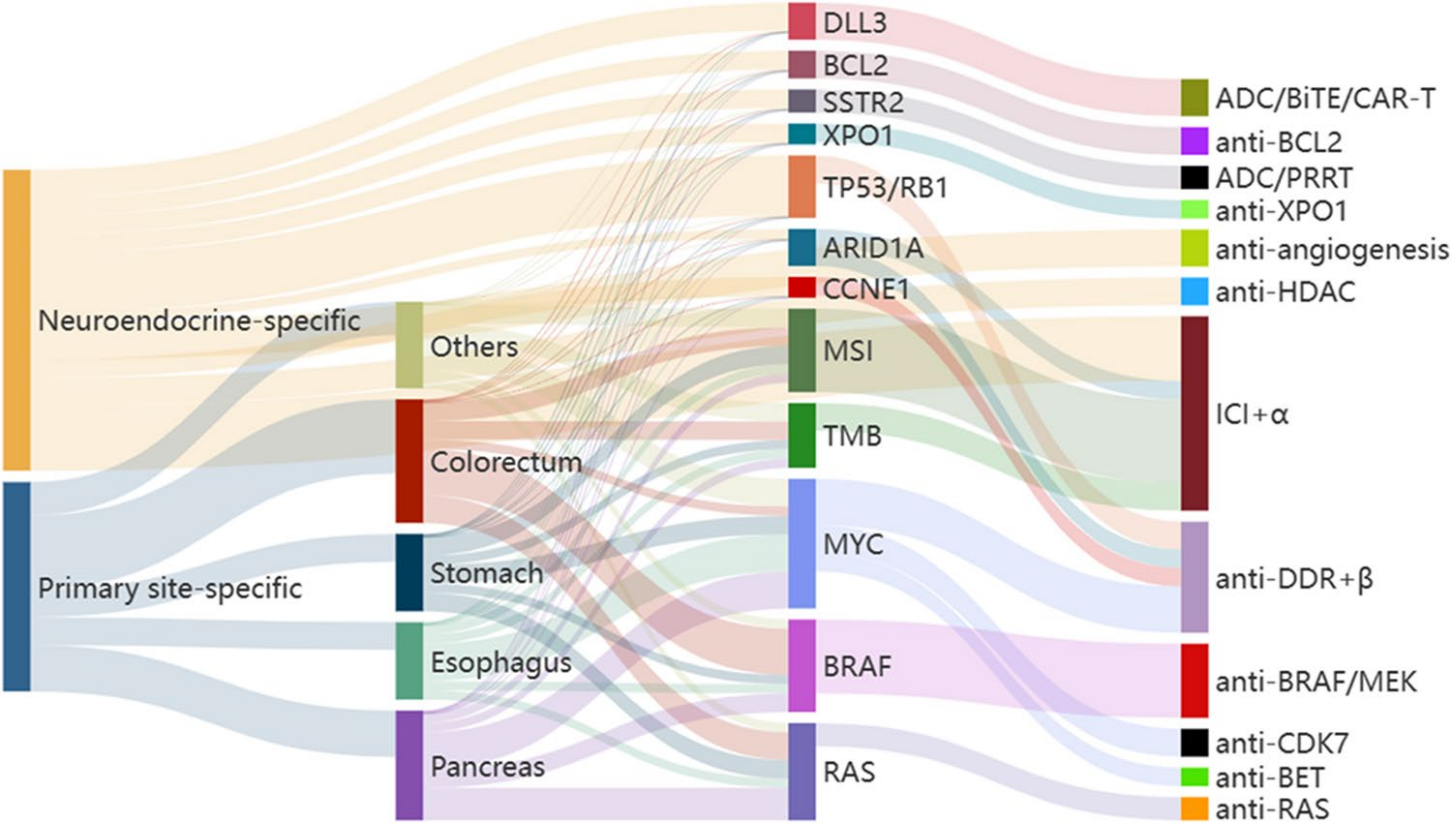
Summary of potent therapeutic strategies for GEP-NEC. The potent approaches are composed of both neuroendocrine-specific and site-specific treatment. ADC, antibody–drug conjugate; BiTE, bispecific T-cell engager; CAR-T, chimeric antigen receptor-T cell therapy; PRRT, peptide receptor radionuclide therapy; HDAC, histone deacetylase; ICI, immune checkpoint inhibitor; DDR, DNA damage response. The “anti- “ means blockade of indicated molecule. The “α” includes other ICIs, chemotherapy, HDAC inhibitor, anti-angiogenic therapy, and DDR inhibitors targeting AURK, WEE1, ATR, ATM, AXL, CHK1, or PARP. The “β” includes other DDR inhibitors, and chemotherapy

Although many molecular-targeted agents are currently approved for various solid tumors, no targeted therapies have been established for the clinical management of NECs. Therefore, novel therapies tailored to their molecular composition are urgently required to improve prognosis. A growing number of comprehensive molecular analyses have provided potential targets for GEP-NEC [10–12, 29, 54, 55, 57], which may lead to therapeutic breakthroughs with a personalized approach. As the molecular landscapes and transcriptional signatures of GEP-NECs are partially similar to those of SCLCs because of the neuroendocrine lineage, treatment strategies for GEP-NEC may be inferred from clinical trials conducted in cases of SCLC, such as immune checkpoint inhibitors (ICIs). As another approach, some molecular aberrations are organ-specific and similar to the corresponding conventional cancer; targeted treatments for conventional cancer may also be indicated for patients with NECs from the same site of origin. Recently, drug sensitivity and gene dependency screens have revealed a common therapeutic vulnerability between SCNECs and hematologic malignant tumors, thus supporting the extrapolation of targeted therapies that have been established for hematologic malignant tumors [4]. In this section, we summarize the results of previous trials (Table 6) and discuss potential therapeutic targets (Fig. 2), as well as other ongoing trials (Table 7).

**Table 6:** Clinical trials of immune checkpoint inhibitors in NEC

Trials	Target	Agent	Line	Phase	Treatment	Primary sites	Key outcomes	Ref
NCT03278405	PD-L1	Avelumab (Avel)	1st	II	Mono	GEP or lung	Among 10 GEP and pulmonary NECs in NET001 cohort, 9 were GEP-NECs. In all patients, ORR, 0%; mPFS, 2.0 months; mOS, 5.7 months	210
NCT03352934			2nd	II	Mono	Any	Among 29 patients with G3 NENs, 16 and 19 were NECs and GEP-G3 NENs, respectively. In an interim analysis, the DCR after 8 weeks was 32% (PR of two); mOS, 4.2 months	213
NCT02939651	PD-1	Pembrolizumab (Pembro)	2nd	II	Mono	Any	Among 29 patients with G3 NEN, 24 and 19 were GEP-NENs and NECs, respectively. In all patients, ORR was 3.4%; mPFS, 8.9 weeks; mOS, 20.4 weeks. Only one patient with a large cell esophageal NEC had an objective PR that was ongoing for 13 months	209
NCT03136055			2nd	II	Mono	Any	Among 14 NECs after failure of previous therapy, 6 were GEP-NECs. In all patients, ORR, 7%; mPFS, 58 days. Because more than 2 of 14 patients did not respond by week 18 in the stage-1 part, the stage-2 part for additional patient enrollment was terminated	211
NCT03136055			2nd	II	Pembro + chemo (CPT-11 or PTX)	Any	Among 22 NECs after failure of previous therapy, 16 were GEP-NECs. Chemotherapy: 17 CPT-11 and 5 PTX. In all patients, ORR, 9%; mPFS, 2 months; mOS, 4 months	244
NCT03167853		Toripalimab (Tori)	2nd	I	Mono	Any	Among 40 NENs (Ki-67 ≥ 10%) after failure of previous therapy, 32 were NECs, including 25 GEP-NECs. ORR, 18.7% in NEC and 25.0% in NET. In all patients, the mPFS and mOS were 2.5 and 7.8 months, respectively	214
NCT03879057			2nd	I	Tori + surufatinib	Any	Among 22 patients with NENs, 14 were NECs. In 13 evaluable NECs, ORR, 23.1%; mPFS, 4.0 months; mOS, 7.5 months	246
NCT04169672			2nd	II	Tori + surufatinib	Any	In 20 evaluable NECs, ORR, 20%; DCR, 70%; mPFS, 3.94 months	247
NCT02955069		Spartalizumab (Spa)	2nd	II	mono	GEP	Among 116 NENs after failure of previous therapy, 21 were GEP-NECs. ORR, 4.8% in GEP-NEC, and 7.4% in NET. In GEP-NEC, mPFS and mOS were 1.8 and 6.8 months, respectively	212
NCT03980925		Nivolumab (Nivo)	1st	II	Nivo + CBDCA + ETP	GEP or UK	Among 38 patients with G3 GEP or unknown NENs in the first-line setting, 26 and 31 were NECs and GEP-NENs, respectively. In all patients, ORR was 54.1%; mPFS, 5.7 months; mOS, 13.9 months. The 12-month OS rate was 58.3% and 54.7% in NETs and NECs, respectively	243
NCT03728361			1st	II	Nivo + TEM	Any	Among 28 NENs, 14 and 8 were GEP-NENs and NECs, respectively. ORR, 38% in NEC and 35% in NET. In the NECs, mPFS and mOS were 6.9 and 32.3 months, respectively	239
NCT03591731			2nd	II (randomized parallel arms)	mono	GEP or lung	Among 91 refractory pulmonary LCNECs or GEP-NECs after failure of previous therapy, 46 were GEP-NECs. ORR at 8 weeks, 7.1% in GEP-NEC, and 7.3% in pulmonary LCNEC. In all patients, the mPFS and mOS were 1.8 and 7.2 months, respectively. The primary endpoint, ORR at 8 weeks, was not met	208
	PD-1/CTLA-4	Nivolumab (Nivo) + ipilimumab (Ipi)	2nd		Nivo + Ipi		Among 94 refractory pulmonary (large-cell only) or GEP-NECs after failure of previous therapy, 46 were GEP-NECs. ORR at 8 weeks, 11.6% in GEP-NEC, and 18.2% in pulmonary LCNEC. In all patients, the mPFS and mOS were 1.9 and 5.8 months, respectively. The primary endpoint, ORR at 8 weeks, was met	208
NCT02834013			2nd	II (basket trial, multiple cohorts)	Nivo + Ipi	Any	Among 32 patients with non-pancreatic NEN, 15 and 18 were GEP-NENs and NECs, respectively. ORR, 44% in NEC and 0% in NET. In all patients, mPFS and mOS were 4 and 11 months, respectively. 6-month PFS rate of 44% in NEC and 14% in NET	234
			2nd		Nivo + Ipi		Among 19 patients with G3 NEN, 9 and 11 were GEP-NENs and NECs, respectively. In all patients, ORR, 26%; mPFS, 2.0 months; mOS, 8.9 months	235
NCT02923934			1st	II	Nivo + Ipi	Any	Among 29 patients with NENs, 6 and 4 were GEP-NETs and GEP-NECs, respectively. ORR, 50% in GEP-NEC and 33% in GEP-NET. In all patients, ORR, 24%; mPFS, 4.8 months; mOS, 14.8 months	236
NCT03095274	PD-L1/CTLA-4	Durvalumab (Durva) + tremelimumab (Treme)	2nd	II (multiple cohorts)	Durva + Treme	GEP	Among 33 patients with GEP-NENs after first line platinum-based chemotherapy, 18 and 15 were GEP-NECs and GEP-G3 NETs, respectively. In all patients, ORR was 9.1%; mPFS, 2.4 months; mOS, 5.9 months. The primary endpoint, the 9-month OS rate, was met	217

Abbreviations: NCT number, ClinicalTrials.gov Identifier; GEP, gastro-entero-pancreatic; NEC, neuroendocrine carcinoma; NEN, neuroendocrine neoplasm; NET, neuroendocrine tumor; UK, unknown; ORR, overall response rate; mPFS, median progression-free survival; mOS, median overall survival; DCR, disease control rate; Ref, reference; Mono, monotherapy

**Table 7:** Ongoing clinical trials of molecular-targeted agents in NEC

Trials	Targets	Agents	Line	Phase	Primary sites	Treatment
mTOR inhibitors						
NCT02113800	mTOR	Everolimus (Eve)	2nd	II	Any	Mono
NCT02687958			1st	II	GEP (Ki-67 < 55%)	Mono
NCT02248012			1st	II	GEP or UK (Ki-67 < 55%)	Eve + TEM
NCT02695459			1st	II	Any	Eve + CDDP
Anti-angiogenetic inhibitors						
NCT04705519	VEGF-A	Bevacizumab (Bev)	2nd	II	Any	Bev + nab-PTX
NCT03457844	VEGFR	Anlotinib (Anlo)	2nd	II	Any	Mono
NCT05165407		Surufatinib (Sur)	2nd	II	Any	Sur + Sin + IBI310
NCT05015621			2nd	III	Any	Sur + Tori vs. FOLFIRI
NCT04412629		Cabozantinib (Cab)	2nd	II	GEP	Mono
NCT04400474			Any	II	Any	Cab + Atezo
NCT04079712			2nd	II	Any	Cab + Nivo + Ipi
Inhibitors of cell-cycle or DDR regulators						
NCT04514497	ATR	Elimusertib (Elimu)	2nd	II	Any	Elimu + CPT-11/TPT
NCT04802174		Berzosertib (Berzo)	2nd	I/II	Any	Berzo + Lurbinectedin
NCT02487095			1st	II	Any (only SCNEC)	Berzo + TPT
NCT03896503			1st	II	Any (only SCNEC)	TPT ± Berzo
NCT04209595	PARP	Rucaparib (Ruca)	2nd	I/II	Any (only SCNEC)	Ruca + PLX038
NCT04701307		Niraparib (Nira)	2nd	II	Any	Nira + Dostarlimab
Inhibitors of epigenetic regulators						
NCT05076786	HDAC	Tucidinostat (Tuci)	1st	II	Extrapulmonary	Tuci + CDDP/CBDCA + ETP
NCT05113355			2nd	II	Any	Tuci + Sin
Immune checkpoint inhibitors						
NCT03147404	PD-L1	Avelumab	2nd	II	GEP	Mono
NCT05058651		Atezolizumab (Atezo)	1st	II/III	Any	CDDP/CBDCA + ETP ± Atezo
NCT05142865	PD-1	Camrelizumab (Camre)	1st	II	Extrapulmonary	Camre + CDDP/CBDCA + ETP + Apatinib
NCT03992911		Toripalimab (Tori)	1st	II/III	Any	Tori + Simmtecan + 5-FU/LV vs. CDDP/CBDCA + ETP
NCT03517488	PD-1/CTLA-4	XmAb20717	2nd	I	Any	Mono
NCT05337735			2nd	II	Any	Mono
DLL3 targeting agents						
NCT04429087	DLL3/CD3	BI 764532	2nd	I	Any (DDL3 +)	Mono
NCT04471727	DLL3/CD3	HPN328	2nd	I/II	Any (DDL3 +)	Mono
SSA/PRRT						
NCT02409849	SSTR	Octreotide-LAR	1st	II	GEP	Mono
NCT00978211		90Y-/177Lu-Dotatate-TOC	Any	II	Any (SSTR2 +)	Mono
NCT04525638		177Lu-Dotatate	1st or 2nd	II	GEP, lung, or UK	Nivo + 177Lu-Dotatate
NCT02936323		PEN-221	2nd	I/Iia	Any (SSTR2 +)	Mono
Others						
NCT02250885	XPO1	Selinexor	2nd	II	GEP, lung, or UK	Mono
NCT05126433	RNA polymerase II	Lurbinectedin	2nd	II	Any	Mono

Abbreviations: NCT number, ClinicalTrials.gov Identifier; GEP, gastro-entero-pancreatic; NEC, neuroendocrine carcinoma; UK, unknown; Mono, monotherapy; TEM, temozolomide; CDDP, cisplatin; CBDCA, carboplatin; nab-PTX, nanoparticle albumin-bound-paclitaxel; Sin, sintilimab (anti-PD-1 Ab); Nivo, nivolumab (anti-PD-1 Ab); Ipi, ipilimumab (anti-CTLA-4 Ab); CPT-11, irinotecan, TPT, topotecan; ETP, etoposide; 5-FU, 5-fluorouracil; LV, leucovorin; SSA, synthetic somatostatin analog; PRRT, peptide receptor radionuclide therapy; SSTR, somatostatin receptor; LAR, long acting release; XPO, exportin-1; Anlotinib, a tyrosine kinase inhibitor that targets VEGFR, FGFR, PDGFR, and KIT; Surufatinib, a small molecule kinase inhibitor of VEGFR1–3, FGFR1, and CSF-1R; Cabozantinib, a small molecule inhibitor of MET, VEGFR, RET, KIT, and the TAM (TYRO3, AXL, MER) family of receptor kinases; IBI310, anti-CTLA-4 Ab; FOLFIRI, 5-fluorouracil + leucovorin + irinotecan; PLX038, pegylated topoisomerase inhibitor SN-38; Dostarlimab, anti-PD-1 Ab; Apatinib, an inhibitor of VEGFR2; XmAb20717, a humanized bispecific monoclonal antibody of PD-1 and CTLA-4; BI 764532, DLL3/CD3 bispecific T-cell engager; HPN328, DLL3/CD3 bispecific T-cell engager

### RAS/BRAF-targeted therapy

Aberrant activation of receptor tyrosine kinase (RTK) pathways is common in non-neuroendocrine epithelial cancers, and *KRAS* mutational activation leads to downstream signaling of the MAPK and PI3K/v-akt murine thymoma viral oncogene homolog (Akt) pathways, which play crucial roles in tumorigenesis, proliferation, survival, angiogenesis, and metastasis. In GEP-NECs, *KRAS* mutations are reported at a similar frequency as in conventional cancers arising at the same sites: *KRAS* genes are frequently mutated in colorectal and pancreatic NECs, while they are less common in esophageal NECs [11, 12] (Table 4). Novel *KRAS G12C* allele-specific covalent inhibitors demonstrated a profound clinical impact in *KRAS G12C*-mutated non-small-cell lung cancer (NSCLC) [143, 144]. The accurate frequency remains unclear in GEP-NEC, but *KRAS G12C* mutations have been observed in NENs [145, 146]. Although *KRAS G12C* mutation may represent a potential target even for GEP-NEC, the efficacy is likely to be contingent upon RTK dependency and signaling rebound kinetics [147, 148]. As there is currently insufficient scientific evidence to establish the therapeutic strategy for *KRAS G12C*-mutated GEP-NEC, further preclinical and clinical studies are needed for GEP-NEC. In addition, the success of targeting *KRAS G12C* will provide hope that a range of mutant *RAS* allele-specific targeted therapies could become therapeutically tractable [149].

An activating missense mutation in codon 600 of exon 15 (*V600E*) of the *BRAF* gene has been identified in various tumor types, and BRAF inhibitors have yielded clinical benefits for patients with *BRAF V600E*-mutated cancers, especially melanoma and NSCLC [150–152]. However, in colorectal adenocarcinoma (CRC), the BRAF inhibitor vemurafenib alone only led to a 5% ORR, indicating insufficient single-agent activity [153]. Based on preclinical findings showing that reactivation of MAPK signaling through feedback activation of epidermal growth factor receptor (EGFR) was an escape mechanism responsible for intrinsic resistance to a BRAF inhibitor alone [154], a combination therapy of the BRAF inhibitor encorafenib and the EGFR inhibitor cetuximab resulted in significantly improved survival in CRC patients with *BRAF V600E* mutation [155]. In melanoma, a combination of the BRAF inhibitor dabrafenib and mitogen-activated protein kinase kinase (MEK) inhibitor trametinib showed superior efficacy over dabrafenib alone [156]. Thus, susceptibility to BRAF inhibitors alone and the success of a combinatorial approach are tumor-lineage-dependent.

*BRAF V600E* mutations are rare events in SCLCs, but the alterations are tumor site-dependent in GEP-NECs and especially enriched in colorectal NECs (Tables 3 and 4). *BRAF* mutations are more frequent genetic events in colorectal NEC than CRC, with frequency ranging from 15 to 59% of colorectal NECs [11, 12, 29, 45, 57, 84–88]. Similar to CRC, the predominant location of *BRAF* mutations is the right side of the colon [12]. EGFR expression is repressed by gene methylation in melanomas, which confers sensitivity to BRAF inhibitors alone [157]. Colorectal NECs have similar EGFR methylation signatures to melanoma, unlike CRC, and BRAF inhibitor monotherapy showed much higher tumor regression in colorectal NECs than CRC in patient-derived xenograft models [88]. In addition, treatment with a dual blockade of BRAF and MEK suppressed cell proliferation and tumor growth by inducing apoptosis and cell cycle arrest at the G1 phase in *BRAF V600E*-mutated colorectal NEC cell lines and xenograft models [87]. In a phase II basket trial of the BRAF inhibitor vemurafenib in non-melanoma *BRAF V600* mutation-positive solid tumors, two NEC patients had PFS of 7.8 months and 5.7 months, respectively [151]. Recently, several case series have reported the benefits of BRAF inhibition in monotherapy or with the addition of an MEK inhibitor in colorectal NECs [84, 88, 158]. Thus, BRAF inhibitors are emerging as the most promising therapeutic strategies for *BRAF V600E*-mutated GEP-NECs (Fig. 2).

### mTOR-targeted therapy

mTOR signaling is aberrantly activated via overexpression of phosphorylated mTOR and dysregulations of the PI3K/Akt pathway, which are implicated in the modulation of cell proliferation, metabolism, and angiogenesis in GEP-NETs [68]. The mTOR inhibitor everolimus showed a significantly prolonged PFS compared to a placebo in GEP-NETs [159], and it has consequently been recommended as a second- or third-line treatment [8, 31, 103]. The PI3K/mTOR pathway is also activated as a recurrent event in GEP-NECs [12, 59, 86]. However, everolimus failed to show efficacy for pancreatic NEC, with an ORR of 0% and median PFS of 1.2 months, in a phase II NECTOR trial [160]. There are ongoing phase II trials of everolimus monotherapy (NCT02113800 and NCT02687958) and a combination of everolimus with temozolomide (NCT02248012) in NEC (Table 7).

### MYC-targeted therapy

MYC is a transcription factor that acts as a master regulator of genes involved in cell cycle progression, cell proliferation, apoptosis, and neuroendocrine lineage plasticity [161, 162]. *MYC* genetic alterations have been frequently reported in GEP-NECs across primary sites [11, 12] (Tables 3 and 4). A pivotal preclinical study demonstrated that MYC overexpression drove trans-neuroendocrine differentiation by binding to neuroendocrine-related genes in genetically engineered pancreatic adenocarcinoma mouse models [161], suggesting a rational target for GEP-NECs (Fig. 2). The direct approach of targeting MYC remains a major clinical challenge due to the unclear structure, absence of intrinsic enzymatic activity, lack of targetable binding pockets, and compensatory activation of the other MYC family members [163]. Therefore, indirect inhibition of MYC is considered an alternative pharmacological approach, such as by targeting its transcription. Cyclin-dependent kinase 7 (CDK7) regulates transcription by affecting the stability of preinitiation complexes, leading to altered gene expression, cell cycle progression, and cell survival [164]. The inhibition of CDK7 has been found to reduce MYC expression by interfering with RNA polymerase II and subsequently inhibiting the super enhancers of MYC [164, 165]. Knockdown or inhibitor treatment of CDK7 showed efficacy in in vitro and in vivo SCLC models [4].

Of note, tumors with MYC aberrations have unique biological vulnerabilities, which represents the potential of precision medicine in these cases [76, 77]. Potent synthetic lethal partners that have preclinically shown promising efficacy in MYC-driven tumors include checkpoint kinase 1 (CHK1), aurora kinase (AURK), WEE1 G2 checkpoint kinase (WEE1), and arginine deprivation. Inactivation of the TP53 and RB1 pathways causes disruption of G1/S cell cycle checkpoint function [42, 43], and MYC activation induces replicative stress, resulting in dependence on G2/M cell cycle checkpoint regulators upon cellular DNA damage [166]. CHK1 is a critical player in regulating the G2/M checkpoint that facilitates cell cycle arrest and DNA damage repair in cells with TP53 aberration [167]. MYC activation is capable of inducing CHK1 overexpression, leading to “CHK1 addiction” in MYC-driven cancers, especially with concurrent inactivation of TP53 [168, 169]. Since GEP-NECs have nearly ubiquitous inactivation of TP53, the CHK1 inhibitor may be more effective in GEP-NECs with *MYC* amplification or overexpression, as shown in cases of SCLC [169]. AURK stabilizes MYC via the regulation of proteasomal degradation mediated by ubiquitin ligases FBXW7 [170, 171]. Stabilized MYC also promotes the transcription of AURK, constituting a positive feedforward loop between MYC and AURK [170]. In a phase II trial of paclitaxel with or without the AURK inhibitor alisertib in SCLC, MYC expression was a predictive biomarker for sensitivity [172]. Thus, MYC-driven cancer cells may be susceptible to AURK inhibitors [173, 174].

Another key component of the G2/M checkpoint is WEE1, which blocks entry into mitosis for proper DNA repair by inhibiting the cyclin-dependent kinase (CDK1 and CDK2) in response to cellular DNA damage [175]. Preclinically, WEE1 inhibition has demonstrated an antitumor effect via cell cycle arrest and apoptosis in SCLC and other neuroendocrine-associated malignancies [176–178], thereby emerging as a therapeutic target for NECs. However, in a biomarker-driven phase II umbrella trial for patients with SCLC after platinum-based chemotherapy, a selective small-molecule WEE1 inhibitor AZD1775 monotherapy showed no objective response in SCLC with *MYC* amplification or co-alterations of *CDKN2A* and *TP53* [179]. Thus, the limited clinical efficacy of WEE1 inhibitor monotherapy suggests an urgent need for novel combination strategies, such as chemotherapy plus AZD1775 for *TP53*-mutant ovarian cancer [180], chemotherapy followed by AZD1775 maintenance for *TP53*/*KRAS*-mutant CRC [181], AZD1775 plus an inhibitor of histone deacetylase (HDAC) or bromodomain-containing protein 4 (BRD4) for acute leukemia [182], or dual blockade of WEE1/AXL receptor tyrosine kinase (AXL) or WEE1/mTOR for SCLC [177].

Metabolic rewiring evokes cellular mechanisms that reduce therapeutic mightiness. Aberration of MYC also leads to reprogramming of cellular metabolism, which creates reliance on arginine biosynthetic pathways, including polyamine biosynthesis and mTOR pathway activation [183, 184]. Arginine depletion with PEGylated arginine deiminase has been found to dramatically suppress the tumor growth of MYC-driven SCLCs in genetically engineered mouse models and a patient-derived xenograft from a relapsed SCLC patient [184]. Depleting arginine may act as a therapeutic strategy for MYC-aberrant GEP-NECs.

While MYC-targeted therapies have emerged as a promising approach for GEP-NEC treatment, they have been hampered due to the lack of available clinical data, highlighting the need for further clinical trials for GEP-NEC.

### DNA damage response-targeted therapy

Recently, targeting components of DNA repair pathways has emerged as a therapeutic strategy [185, 186]. DNA damage response (DDR) pathways play a critical role in cell survival through the activation of DNA repair signaling and their interaction with cell cycle checkpoints [185]. In cancer, DDR pathways are frequently disrupted by alterations in DDR-related genes, causing genomic instability as one of the hallmarks of cancer [186]. The poly (ADP-ribose) polymerase (PARP) enzyme acts as a highly sensitive sensor for DNA damage, which recruits DNA repair proteins to damage sites to facilitate efficient repair [185]. PARP is activated in response to DNA double-strand breaks (DSBs), but DSBs are normally repaired by homologous recombination repair (HRR). Cancer cells with HRR deficiency rely on an alternative repair system mediated by PARP, leading to susceptibility to PARP inhibitors via enhanced synthetic lethality due to a blockade of the repair system [187]. The clinical success of PARP inhibitors in *BRCA*-mutated breast, ovarian, prostate, and pancreatic cancers has provided proof-of-concept for synthetic lethality as a novel therapeutic strategy. DDR pathways are also sometimes deficient in SCLC and extrapulmonary NEC [188, 189]. In addition, the targeted sequencing in 152 GEP-NEC samples showed that the majority of potentially targetable alterations were related to defects in DNA repair [12]. In a randomized phase II trial of the PARP inhibitor veliparib in combination with EP chemotherapy in extensive-stage SCLC, the PFS as a primary endpoint was met, with an improvement in median PFS of 6.1 months for veliparib versus 5.5 months for the placebo [190]. A phase I/II trial of the PARP inhibitor rucaparib plus a PEGylated conjugate of SN-38 (PLX038) acting as a DNA-damaging chemotherapy is ongoing in solid tumors and small-cell cancers, including GEP-NEC (NCT04209595) (Table 7). The combined blockade of DDR proteins, such as PARP and WEE1, may also enhance therapeutic efficacy because of their crosstalk [186, 191].

In addition to PARP, DDR kinases, such as ataxia telangiectasia and RAD3-related (ATR), ATM, CHK1, and WEE1, have emerged as attractive targeted molecules because of their central roles in DNA repair [192, 193]. ATR is activated by DNA damage or DNA replication stress, which not only stabilizes replication forks but also activates the G2/M checkpoint. A subset of cancer cells under replication stress may be susceptible to ATR inhibitors, as well as other DDR inhibitors, such as CHK1 [168, 169] or WEE1 [177, 180] under MYC- or CCNE1-induced replication stress. Based on the preclinical results indicating that the dual inhibition of ATR and topoisomerase I was synergistically cytotoxic in SCLC, a proof-of-concept phase II trial of the ATR inhibitor berzosertib plus topotecan was conducted in the second or latter line of SCLC, which showed an ORR of 36% and a median PFS of 4.8 months [194]. Given that extrapulmonary SCNECs share a common molecular profile with SCLC [4, 5, 30], the trial was amended to assess the efficacy of berzosertib plus topotecan in extrapulmonary SCNECs. In this case, the ORR was 20% in 10 patients with extrapulmonary SCNECs from distinct primary sites, including the GEP system, and responses were observed even in tumors refractory to prior treatment with the topoisomerase I inhibitor [194]. In an exploratory analysis using pre-treatment samples from both SCLC and extrapulmonary SCNEC, most responders exhibited high neuroendocrine differentiation, such as ASCL1 or NEUROD1 subtypes, and somatic copy number alterations in genes driving replication stress, including CCNE1 gain and ARID1A loss [194]. Collectively, exacerbating DNA replication stress may induce the therapeutic vulnerability of GEP-NECs to DDR inhibitors (Fig. 2). Currently, several early-phase trials of agents targeting ATR in combination with a topoisomerase I inhibitor (NCT04514497, NCT02487095, and NCT03896503) are ongoing in cancers, including GEP-NECs (Table 7).

### Angiogenesis-targeted therapy

Most pancreatic NETs have an extraordinary tumor vascularization due to overexpression of pro-angiogenic factors, including vascular endothelial growth factor receptor (VEGFR) and platelet-derived growth factor receptor (PDGFR) [195]. In fact, the multi-targeted tyrosine kinase inhibitor (TKI), mainly targeting VEGFRs and PDGFRs, sunitinib [196], and the mTOR inhibitor everolimus [159] have proven to be of clinical benefit in pancreatic NETs, and the promising antitumor activity of anti-angiogenic TKIs, such as pazopanib [197], cabozantinib [198], lenvatinib [199], and surufatinib [200, 201], has also been demonstrated in NETs.

Although there are no approved anti-angiogenetic agents for NECs, a preclinical study has demonstrated potent antitumor activity for two anti-VEGF antibodies, bevacizumab and aflibercept, in xenograft models of SCLC and colon NEC cell lines [202]. Clinically, a retrospective study reported the potent efficacy of the anti-VEGFR2 antibody ramucirumab in combination with chemotherapy compared to chemotherapy alone, possibly due to high expression levels of VEGFR2 in metastatic gastric NEC [203]. In a randomized phase II PRODIGE41-BEVANEC trial of bevacizumab in combination with second-line 5-FU, leucovorin, and CPT-11 (FOLFIRI) after the failure of a platinum plus ETP regimen in patients with GEP-NECs, the primary endpoint was met, with ≥ 50% of patients alive at 6 months following treatment with FOLFIRI plus bevacizumab. However, there seemed to be no additional efficacy of bevacizumab when added to FOLFIRI, given the median OS of 8.9 months and 7.0 months in FOLFIRI alone versus FOLFIRI plus bevacizumab, respectively [204]. In a phase II trial of first-line capecitabine, oxaliplatin, and CPT-11 (CAPOXIRI) plus bevacizumab, followed by maintenance treatment with pazopanib plus capecitabine, for colon or small intestinal NECs, great efficacy was shown, with an ORR of 47.4%, median PFS of 13 months, and median OS of 29 months [205]. In addition, phase II trials in cases of GEP-NEN showed clinical activity of sunitinib, with a disease control rate (DCR) of 55% in 20 patients with GEP-NEC [206], and pazopanib with an ORR of 23% and median PFS of 5.8 months in 13 patients with GEP-NEC [207]. There are ongoing trials of agents targeting angiogenic molecules in cancers, including GEP-NECs: a phase II trial of bevacizumab plus nab-paclitaxel in NEC (NCT04705519), a phase II trial of multi-targeted TKI (mainly targeting VEGFRs and PDGFRs), anlotinib in G3 GEP-NET including NEC (NCT03457844), and a phase II trial of cabozantinib targeting VEGFR2/MET proto-oncogene/AXL in G3 NENs including NEC (NCT04412629) (Table 7 and Fig. 2).

### DLL3-targeted therapy

Delta-like canonical Notch ligand 3 (DLL3) is an inhibitory ligand of the Notch receptor pathway and is highly expressed in most pulmonary NECs [208, 209], which drives neuroendocrine differentiation [42]. DLL3 is a downstream target of ASCL1 [210], which acts as a prominent transcription factor in GEP-NECs [56, 58, 78]. In fact, DLL3 and ASCL1 have been molecularly and clinically characterized as the same subgroup among extrapulmonary NECs [78]. DLL3 was frequently expressed in 76.9% of GEP-NECs, but not in G1-G3 NETs [211]. In addition, DLL3 was found to be differentially upregulated in esophageal NEC compared to matched normal esophagi, accounting for approximately 85% of esophageal NECs [58]. Considering the high prevalence of NEC-specific cell surface molecules, DLL3 could be a compelling therapeutic opportunity for an antigen targeted by antibody–drug conjugates (ADCs), bispecific T-cell engager (BiTE), and chimeric antigen receptor (CAR) T cells in GEP-NECs (Fig. 2).

DLL3 is an attracted molecule that delivers cytotoxic compounds selectively and directly to NEC cells. Rovalpituzumab tesirine is an ADC comprising the cytotoxic payload pyrrolobenzodiazepine, which is conjugated by a linker to a monoclonal DLL3 antibody. Despite promising preclinical and early-phase clinical antitumor activity [208, 210], phase III trials of DLL3-positive SCLC have failed to demonstrate significantly improved OS of rovalpituzumab tesirine as a maintenance therapy versus a placebo after platinum-based therapy [212] and as a second-line treatment versus topotecan [213]. In a phase I/II trial of DLL3-expressing solid tumors, including GEP-NECs, patients with NEC/NET had an ORR of 13%, with a median PFS of 4.1 months [214]. These results led to the discontinuation of the further development of rovalpituzumab tesirine. The development of another DLL3-targeting ADC with cytotoxic pyrrolobenzodiazepine, SC-002, was also discontinued because of systemic toxicity [215]. However, the toxicity profiles of both rovalpituzumab tesirine and SC-002 were attributed to the cytotoxic pyrrolobenzodiazepine, suggesting that DLL3 still remains a research interest as a target of ADC.

Tarlatamab (AMG 757) is a half-life extended BiTE designed to specifically bind DLL3 on cancer cells and CD3 on T cells, resulting in T-cell-dependent killing of cancer cells with DLL3 expression in the SCLC patient-derived xenograft models [216]. A phase I trial of tarlatamab showed a confirmed ORR of 13%, with the estimated duration of response ≥ 6 months in 71% of cases of SCLC [217]. BI 764532 is a novel IgG-like DLL3/CD3 BiTE, resulting in T-cell-mediated complete tumor regression in a human T-cell engrafted xenograft model [218]. A first-in-human phase I trial of BI 764532 is ongoing in patients with SCLC and other NECs expressing DLL3 (NCT04429087). HPN328 is a tri-specific T-cell-engager designed as three binding domains with anti-albumin for half-life extension in addition to DLL3/CD3. Interim results of an ongoing phase I/II trial (NCT04471727) of HPN328 showed any tumor shrinkage in 40% of 15 patients with SCLC and other NECs expressing DLL3 [219]. Treatment with DLL3-targeted CAR-T cells also resulted in preclinical antitumor activity in SCLC xenograft models [220], and the DLL3-targeting CAR-T cells AMG 119 are in clinical development [221]. Thus, DLL3-targeted products may lead to a tremendous breakthrough in treating GEP-NECs (Table 7).

### Epigenetic-targeted therapy

Epigenetic alternations, including DNA methylation, histone acetylation, and histone methylation, regulate gene expression and interact with numerous transcription factors that have fundamental functions in cancer progression [222]. The enhancer of the zeste 2 polycomb repressive complex 2 subunit (EZH2) is an enzymatic catalytic subunit of the polycomb repressive complex that can epigenetically alter gene expression via histone methyltransferase [223]. EZH2 overexpression and specific methylation patterns frequently occur in SCLC and other NECs, including the GEP system, which contribute to cellular lineage plasticity [10, 48, 49, 189]. DNA-damaging chemotherapy was found to induce genome-wide EZH2 activity, which in turn drove chemoresistance through epigenetically silencing the cell cycle regulator Schlafen family member 11 (SLFN11) [224]. The addition of EZH2 inhibitors to cytotoxic chemotherapy prevented the emergence of acquired resistance and augmented chemotherapeutic efficacy in both chemosensitive and chemoresistant SCLC patient-derived models. Although a phase I trial of selective EZH2 inhibitor PF-06821497 monotherapy failed to show a treatment response in two SCLC patients [225], these preclinical findings provide a rationale for further development of epigenetic targeting strategies.

Bromodomain and extra-terminal (BET) proteins bind acetylated histones and recruit protein complexes to promote transcription, among which BRD4 serves as a transcriptional regulator of MYC [226, 227]. BET inhibitors preclinically impaired tumor growth in MYC-dependent cancers, including SCLC [227, 228]. In addition, ASCL1 was downregulated by binding the BET inhibitor to the ASCL1 enhancer [229]. Of note, hematologic malignant tumors have been found to have more similarities to SCNECs in terms of expression profiles and drug sensitivity-based phenotypes, and SCNECs are more sensitive to HDAC inhibitors approved in hematologic malignancies [4]. To evaluate the additive and potentially synergistic effects of combining an HDAC inhibitor with chemotherapy, a phase I study of the HDAC inhibitor belinostat in combination with EP chemotherapy was conducted in advanced solid cancer [230]. In 15 patients with NECs, including 4 GEP-NECs, the ORR and DCR were 47% and 93%, respectively. A phase II trial of a novel subtype-selective HDAC inhibitor, tucidinostat, in combination with chemotherapy is ongoing in NECs (NCT05076786) (Table 7 and Fig. 2).

### Somatostatin-targeted therapy

Somatostatin receptors (SSTRs) are a family of G protein-coupled receptors that are implicated in the regulation of hormone secretion and tumor proliferation in NET [231]. Since SSTRs are frequently expressed in NET, SSAs, such as octreotide and lanreotide autogel, have been established as a first-line therapy for ameliorating secretory symptoms and tumor growth in patients with SSTR expressing NET [232–234]. PRRT is a radiolabeled SSA, conjugated with a chelator and β/γ-emitting 177Lutetium (^177^Lu) or β-emitting 90Yttrium (^90^Y), in order to kill neoplastic cells with lethal radiation [235]. A phase III NETTER-1 trial showed significantly improved PFS and ORR in PRRT with [232]Lu labeled-tetraazacyclododecanetetraacetic acid (DOTA) modified Tyr [3] octreotate (TATE) compared to high-dose octreotide long-acting release (LAR) for patients with midgut NETs [236]. Based on this trial, PRRT is a second-line therapeutic option for GEP-NETs.

Although NECs have generally absent or reduced SSTR expression [1, 7, 16, 30, 31], a subset of NECs presents SSTR expression, especially for LCNECs and NECs, with a Ki-67 value of 21–55% [41, 91, 237]. In a multicenter retrospective cohort of 149 patients with G3 GEP-NENs treated with PRRT, the median PFS was 19 months in G3 NET, 11 months for NEC with Ki-67 ≤ 55%, and 4 months for NEC with Ki-67 > 55% [237, 238]. Of note, the ORR was similar between the NECs with Ki-67 ≤ 55% and > 55% (43% vs. 46%), and the median OS was 9 months, even in NECs with Ki-67 > 55%. PRRT may thus be considered a promising therapeutic option for selected NEC patients [7, 238]. Currently, several phase II trials have investigated the treatment efficacy of SSAs or PRRT in GEP-NECs, including octreotide LAR (NCT02409849), PRRT with [145]Y-DOTA modified somatostatin analog Tyr [3]-octreotide (TOC) and [232]Lu-DOTA-TOC (NCT00978211), and a combination of PRRT with [232]Lu-DOTA-TATE and the anti-PD-1 antibody nivolumab (NCT04525638) (Table 7 and Fig. 2).

SSTR2 is one of the most frequently expressed subtypes of SSTRs in GEP-NENs. PEN-221 is a small peptide drug conjugate that selectively targets SSTR2, with a cleavable linker to a cytotoxic payload DM1. Treatment with PEN-221 was found to enable efficient drug delivery to SSTR2-positive cells, resulting in complete and durable regressions in SSTR2-positive SCLC xenograft mouse models [239]. A phase I/IIa trial assessed the preliminary antitumor activity and safety of PEN-221 in patients with SSTR2-expressing NENs, including GEP-NEC (NCT02936323) (Table 7 and Fig. 2).

### Other potent molecular-targeted therapies

Other potential cellular targets in GEP-NECs have been identified, such as exportin-1 (XPO1), the BCL2 apoptosis regulator (BCL2), and lurbinectedin (Fig. 2).

XPO1 is a key nuclear export protein that regulates the nucleocytoplasmic trafficking of a growing number of tumor suppressor proteins, growth regulatory proteins, and chemotherapeutic agents [240, 241]. XPO1 aberration leads to the functional inactivation of tumor suppressor proteins through exportation from the nucleus to the cytoplasm via the nuclear pore complex, which is implicated in tumorigenesis in various tumor types [241]. In a screening of potential therapeutic vulnerabilities using clustered regularly-interspaced short palindromic repeats (CRISPR)/CRISPR-associated protein (Cas)9 technology in SCLC cell lines, XPO1 was identified as a promising target for CDDP sensitization [242]. Selinexor is a selective inhibitor of nuclear export compounds that forms a reversible covalent bond with the cysteine residue of the XPO1 cargo-binding pocket, leading to nuclear retention and functional activation of tumor suppressor proteins and hindering DDR mechanisms [243]. Selinexor has been granted U.S. Food and Drug Administration (FDA) approval for the treatment of multiple myeloma and diffuse large B-cell lymphoma, and synergistic effects between selinexor and DNA-damaging agents have been preclinically demonstrated in cases of SCLC [242]. A phase II trial also investigated the efficacy of selinexor in SCLC and GEP-NEC (NCT02250885) (Table 7).

BCL2 plays an important role in blocking apoptotic cell death [244]. Accordingly, BCL2 was identified as a druggable target with conserved expression across the site of origin in NECs [176]. BCL2 inhibitors have demonstrated remarkable clinical benefit in hematologic malignant tumors and have been included in the shared predicted drug sensitivity profiles between hematologic malignancies and SCNECs [4]. SCLC cells with the ASCL1 molecular subtype predominantly exhibited BCL2 overexpression, and they were sensitive to BCL2 inhibitors [173, 245]. BCL2 inhibitors may also promote synergistic antitumor activity in combination with WEE1 inhibitors or BET inhibitors in cases of NECs [176, 246]. In GEP-NECs, BCL2 overexpression has also been observed at a high prevalence, partially due to the predominance of ASCL1 [9, 63], thus indicating a potential therapeutic target.

Lurbinectedin is a selective inhibitor of oncogenic transcription through preferential binding to CpG-rich sequences around promoters of protein-coding genes, degradation of elongating RNA polymerase II, generation of DNA damage, and subsequent apoptosis [247]. Lurbinectedin abrogates the expression of ASCL1 and NEUROD1 transcription factors and their target genes, such as BCL2, INSM1, MYC, and AURK in SCLC [248], which has been approved as a second-line therapy of SCLC by the FDA, with an ORR of 35% in patients with relapsed SCLC [249]. In a cohort of NEN patients from a phase II basket trial of lurbinectedin, two of the 31 evaluable participants had confirmed PR, and one patient with PR was diagnosed with NEC [250]. The efficacy of lurbinectedin monotherapy was assessed in a phase II trial that included a cohort of patients with NEC (NCT05126433). In a drug screening of lurbinectedin in combination with 43 other agents in SCLC, the top synergistic agent was the ATR inhibitor berzosertib, with a 3.5-fold increase in DNA damage compared to lurbinectedin alone [251]. Currently, a phase I/II trial of lurbinectedin plus the ATR inhibitor berzosertib is ongoing in NECs (NCT04802174) (Table 7).

Liquid biopsy has attracted considerable attention as a less-invasive approach that can identify high-level and clonal alterations among tumors with intratumoral heterogeneity [252]. The feasibility of next-generation sequencing using circulating tumor DNA (ctDNA) has also been demonstrated in GEP-NECs [253–255], suggesting the potential to provide precision medicine for patients with more homogenous alterations in the near future.

### Immunotherapy

Inhibitory immune checkpoint molecules, such as programmed death-1 (PD-1) and its ligand PD-L1, promote antitumor immune escape during the cancer–immunity cycle process [256, 257]. Clinically, ICIs targeting PD-1/PD-L1 have exhibited a durable response by disrupting immune tolerance and activating cytotoxic T cells in various tumor types. While ICIs already constitute a standard treatment modality for patients with SCLC [258], ICI monotherapy has limited antitumor efficacy [77, 259–262]. The clinical benefit of ICIs has been evaluated for patients previously treated for extrapulmonary NECs in several early trials (Table 6). Consistent with SCLC, ICI monotherapy was unfortunately less effective in the unselected populations of GEP-NECs, with an ORR of 0–18.7% [262–268]. PD-L1 expression was associated with high-grade classification in NENs [266, 269, 270], but the association between PD-L1 expression and the treatment efficacy of ICI remains controversial [263, 266, 268, 271]. PD-L1 expression is commonly weak and restricted to tumor-infiltrating lymphocytes (TILs) rather than tumor cells in GEP-NECs [69, 269]. TILs have also been found to be abundant in GEP-NEC compared to GEP-NET [270, 272], but usually at a low density and located at the tumor edges or at the surrounding stroma without infiltrating the tumor parenchyma [58, 273]. In addition, adaptive immunity in a subset of NEC cases was counteracted by immune escape mechanisms, such as loss of major histocompatibility complex (MHC) class I, and by negative regulation of adaptive immunity via cyclooxygenase-2 and β-catenin signaling [270]. In a systemic review and meta-analysis of ICIs in 464 patients with NENs, the ORR was higher in NECs versus NETs and in the ICI combination versus monotherapy, although GEP-NENs had lower ORRs than pulmonary NENs [274]. Collectively, these findings likely indicate the limited potential of ICI monotherapy in GEP-NECs. Therefore, there is an urgent need for improved biomarkers for patient selection and the identification of synergistic therapeutic combinations.

Based on the clinical benefit of anti-PD-1 Ab pembrolizumab for patients with microsatellite instability-high (MSI-H)/deficient mismatch repair (dMMR) tumors in pivotal clinical trials [275], the FDA granted first tumor-agnostic approval for pembrolizumab for MSI-H/dMMR tumors. The frequency of MSI-H has been reported as ranging between 0 and 13% of GEP-NECs [12, 27, 52, 54, 66, 69, 70]. Like the corresponding conventional adenocarcinoma at the site of origin [71–73], MSI-H has been found to predominantly have small intestinal, gastric, and colorectal origins among GEP-NECs, mostly subsequent to MHL1 promoter methylation [11, 54, 66, 70]. The tumor mutational burden (TMB), defined as the total number of mutations per coding area of a tumor genome, is an emerging biomarker response to pembrolizumab [276], which led the FDA to approve it for patients with TMB-high tumors (≥ 10 mutations/megabase). TMB-high status is also more pronounced in high-grade GEP-NENs [52], but an analysis of a diverse cohort of 100,000 cancer cases showed a TMB-high designation in only 1.7–8.5% of extrapulmonary NECs, depending on primary tumor sites [277]. In addition, the prevalence of TMB-high and median values of TMB is generally lower in GEP-NECs than in SCLCs [11, 12, 42, 52, 53, 58, 69, 277]. *ARID1A* is a more common altered gene in GEP-NEC [10–12, 52]. ARID1A is a subunit of SWI/SNF chromatin remodeling complex, and its aberration induces the dysregulation of transcription, DNA repair, and chromatin segregation [278]. ARID1A deficiency impairs the MMR system, resulting in an increased MSI-H genomic signature, TMB, TILs, and PD-L1 expression [279]. Preclinically, ARID1A-deficient tumors, but not ARID1A-wild-type tumors, were regressed by treatment with ICI in xenograft models [279]. Clinically, in a phase I trial of NENs, in which 80% of patients had NECs, 3 of 8 (37.5%) responders to anti-PD-1 Ab toripalimab had *ARID1A* mutations, while only one of 27 non-responders harbored mutations [268]. Thus, a subset of GEP-NEC with MSI-H, TMB-high, or ARID1A deficiencies may preferentially benefit from ICIs (Fig. 2) [268].

Several treatment strategies have been examined to turn immunologically “cold” tumors with poor immune activation into “hot” tumors with strong immune infiltration in clinical trials combining the anti-PD-1/PD-L1 antibody with other immune-modulating treatments, including other ICIs, chemotherapy, angiogenetic inhibitors, and molecular-targeted agents (Table 7 and Fig. 2). Currently, the most promising strategy for the ICI combination is a dual blockade of PD-1 and cytotoxic T-lymphocyte-associated antigen-4 (CTLA-4). CTLA-4 acts as a negative regulator of the initial priming of T cells in the early stage of the immune response process, whereas PD-1/PD-L1 acts in later stages by turning off antitumor T-cell responses [280]. Therefore, dual inhibitors synergistically promote an antitumor immune response by blocking complementary mechanisms. In SCLC, a combination of anti-PD-1 Ab nivolumab plus anti-CTLA-4 Ab ipilimumab showed more favorable ORRs than nivolumab monotherapy, but the combined regimen was more toxic and similar OS to nivolumab monotherapy [259]. In a phase III CASPIAN trial of anti-PD-L1 Ab durvalumab with or without anti-CTLA-4 Ab tremelimumab in combination with first-line platinum-based chemotherapy, durvalumab plus chemotherapy demonstrated additional survival benefits compared to chemotherapy alone, whereas the addition of tremelimumab to durvalumab plus chemotherapy did not significantly improve outcomes [281]. The efficacy of anti-CTLA-4 Ab may be enriched in patients with TMB-high status [282]. The initial report of the dual inhibition of PD-1/PD-L1 and CTLA-4 was from a phase II multi-cohort trial of nivolumab plus ipilimumab in 32 patients with non-pancreatic NEN, where all responders were observed in an NEC cohort, with an ORR of 44% and a 6-month PFS rate of 44% [283]. Among 8 patients with GEP-NEC, two patients had tumor response. Subsequently, clinical activity was reported in several phase II trials for NENs, including cases of GEP-NEC, ranging from 9 to 50% in terms of ORR [262, 271, 284, 285] (Table 6). The antitumor efficacy of ICIs, including bispecific monoclonal antibodies of PD-1 and CTLA-4, XmAb20717 (NCT05337735 and NCT03517488), as well as anti-PD-L1 Ab avelumab monotherapy (NCT03147404), is currently under investigation in NECs (Table 7).

The PD-1/PD-L1 interaction is not the only immune checkpoint pathway that regulates T-cell activation in the tumor microenvironment (TME). Lymphocyte activation gene 3 protein (LAG3), T-cell immunoglobulin mucin receptor 3 (TIM3), and T-cell immunoreceptor with Ig and ITIM domains (TIGIT) are overexpressed on effector CD4 + and CD8 + T cells, regulatory T cells (Tregs), and natural killer cells, which act as inhibitory immune checkpoint modulators [286]. In fact, TIM3 and LAG3 in immune cells likely hamper the response to ICIs in NECs [287, 288]. TIGIT binds to CD155 with high affinity and competes with its activating counterreceptor CD226, which contributes to the local suppression of immune surveillance. A preclinical model showed that dual blockade of TIGIT and PD-L1 synergistically and specifically enhanced CD8 + T-cell effector function [289]. However, in a phase III SKYSCRAPER-02 trial of anti-PD-L1 Ab atezolizumab plus first-line chemotherapy with or without anti-TIGIT Ab tiragolumab in SCLC, tiragolumab did not provide an additional survival benefit [290]. Further studies of the ICI combination are required for NEC.

Chemotherapy can promote immune responses by increasing the immunogenicity of cancer cells or inhibiting immunosuppressive circuitries [291]. The clinical benefits of anti-PD-1/PD-L1 Ab in combination with first-line chemotherapy for patients with SCLC have been demonstrated [258, 281]. In a phase II NICE-NEC trial of nivolumab plus first-line platinum-based chemotherapy for 38 patients with unresectable G3 NENs of GEP or unknown origin, including 26 patients with NEC, nivolumab plus chemotherapy showed promising activity, with a 12-month PFS rate of 17.5%, a 12-month OS rate of 53.8%, and an ORR of 54% [292]. The median OS seemed to vary according to the primary site, with 6.4 months being reported for colorectal NENs and not reached for esophagogastric and small intestinal NENs. In another study, pembrolizumab plus chemotherapy failed to demonstrate treatment efficacy for 22 patients with previously treated extrapulmonary NECs, including 16 patients with GEP-NECs, with an ORR of 9% and a median PFS of 2 months [293]. The therapeutic strategy combining ICIs with chemotherapy is currently under investigation in several trials (NCT05058651, NCT05142865, and NCT03992911) (Table 7).

As the VEGF/VEGFR signaling pathway induces immunosuppressive effects via the downregulation of MHC expression, the activation of inhibitory immune checkpoint molecules, and the inhibition of TILs and dendritic cell differentiation in addition to angiogenesis [257], the combination of anti-angiogenetic agents with ICI has emerged as a promising strategy with immunomodulatory effects. In fact, the most compatible partners of ICIs have been found to be anti-angiogenetic inhibitors and platinum chemotherapy in a cross-sectional study of 98 clinical trials that included 24,915 patients [294]. Surufatinib is a small-molecule kinase inhibitor that primarily acts on VEGFR 1, 2, and 3; fibroblast growth factor receptor 1 (FGFR 1); and colony-stimulating factor 1 receptor (CSF-1R). Among 13 evaluable patients with NEC in a phase I trial of surufatinib plus toripalimab for advanced solid tumors, the ORR and median PFS were 23.1% and 4.0 months, respectively [295]. In an NEC cohort of the subsequent phase II trial, similar results were observed, with an ORR of 20% and a median PFS of 3.9 months [296], which was a favorable result compared to those of previous trials of ICI monotherapy in NECs (Table 6). Currently, a phase III SURTORI-01 trial (NCT05015621) is ongoing to evaluate the efficacy of surufatinib plus toripalimab versus FOLFIRI chemotherapy in a second-line setting for patients with NEC. In addition, there are several ongoing trials combining anti-PD-1/PD-L1 Ab with surufatinib plus anti-CTLA-4 Ab IBI310 (NCT05165407), cabozantinib (NCT04400474), and cabozantinib plus ipilimumab (NCT04079712) (Table 7).

HDAC inhibitors have been found to enhance tumor immunogenicity through not only increased MHC presentation [297] but also the reduced number and function of myeloid-derived suppressor cells and Tregs [298, 299]. On the other hand, treatment with HDAC inhibitors resulted in the upregulation of PD-L1 [300], which provides a rationale for combining HDAC inhibitors with anti-PD-1/PD-L1 Ab. In fact, early clinical trials have shown encouraging effects of the combined treatment in lung cancer and head and neck cancer [301, 302], and a phase II trial of tucidinostat plus anti-PD-1 antibody sintilimab is ongoing in advanced G3 NENs (NCT05113355).

The DDR pathway is sometimes deficient in NECs [12, 188, 189], which may contribute to the efficacy of ICIs through increased mutation load and neoantigen burden due to the loss of normal DNA repair function [303]. Thus, the DDR pathway and immune responses are connected and potentially synergistic, and combined treatment with ICI and DDR inhibitors, therefore, may have the potential to reinforce antitumor immune activity. As PARP inhibitors also promote host immunosuppression by upregulating PD-L1 expression, a dual blockade of PARP and PD-1/PD-L1 may be a relevant strategy to induce greater antitumor efficacy than inhibition alone [304, 305]. A phase II trial of the PARP inhibitor niraparib plus anti-PD-1 Ab dostarlimab is currently being conducted for SCLC and other NECs (NCT04701307). In addition, CHK1 [304], CDK7 [306], and WEE1 [178] have been shown to be compatible partners of ICIs in preclinical studies of SCLC.

## Conclusion

NEC is a rare histological subtype among cancers in the GEP system, and the unsatisfying prognosis highlights the clinically urgent need for effective therapeutic compounds. NEC partially shares molecular features specific to SCLC across primary organ sites, whereas NEC also has key genetic aberrations similar to the non-neuroendocrine conventional cancer arising in the same organ sites. Thus, the molecular landscape of GEP-NECs is composed of both neuroendocrine-specific and site-specific alterations, indicating that there is potential in the extrapolation of effective treatment strategies, not only from SCLC but also from conventional cancers at the same site of origin. A better understanding of GEP-NEC biology could reveal a population vulnerable to specific molecular inhibition, which may pave the way for the establishment of personalized medicine. In addition, the assessment of ctDNA will guide the selection of patients who may benefit from molecular-targeted agents by identifying clonally altered genes in GEP-NECs with intratumoral heterogeneity.
